# Changes in MDA5 and TLR3 Sensing of the Same Diabetogenic Virus Result in Different Autoimmune Disease Outcomes

**DOI:** 10.3389/fimmu.2021.751341

**Published:** 2021-11-05

**Authors:** Pamela J. Lincez, Iryna Shanina, Marc S. Horwitz

**Affiliations:** ^1^Michael Smith Laboratories, The University of British Columbia, Vancouver, BC, Canada; ^2^Department of Microbiology & Immunology, Life Sciences Institute, The University of British Columbia, Vancouver, BC, Canada

**Keywords:** autoimmunity, diabetes, interferon, MDA5, TLR3, coxsackievirus, interferonopathy

## Abstract

Seemingly redundant in function, melanoma differentiation-associated protein 5 (MDA5) and toll-like receptor- 3 (TLR3) both sense RNA viruses and induce type I interferon (IFN-I). Herein, we demonstrate that changes in sensing of the same virus by MDA5 and TLR3 can lead to distinct signatures of IFN-α and IFN-ß resulting in different disease outcomes. Specifically, infection with a diabetogenic islet β cell-tropic strain of coxsackievirus (CB4) results in diabetes protection under reduced MDA5 signaling conditions while reduced TLR3 function retains diabetes susceptibility. Regulating the induction of IFN-I at the site of virus infection creates a local site of interferonopathy leading to loss of T cell regulation and induction of autoimmune diabetes. We have not demonstrated another way to prevent T1D in the NOD mouse, rather we believe this work has provided compounding evidence for a specific control of IFN-I to drive a myriad of responses ranging from virus clearance to onset of autoimmune diabetes.

## Introduction

As is commonly described for most autoimmune diseases, type 1 diabetes (T1D) is a disease that results from changes in specific genes, disruption in the balance of immune responses, and exposure to environmental agents like viruses (1–3). With studies and data that continue to emerge from major T1D pancreatic tissue biobanks (4, 5), it is increasingly clear that genetics alone cannot account for the beta cell destruction and insulitis that ensues in the pancreas of people (mostly children) suffering with T1D.

Although not included as type 1 interferonopathies, organ-specific autoimmune diseases such as T1D have been strongly associated with upregulation of the type 1 interferon (IFN-I) response. In children at risk for T1D, an IFN-I transcriptional signature precedes islet autoimmunity (6). In recent onset studies, patients with insulitis-affected islets have an overexpression of interferon-stimulated genes (ISGs), comparable to responses seen in islets infected with virus or treated with inflammatory agents like IFN-α or IFN-γ (7, 8). Included in the overexpressed ISGs identified in T1D patients, are the genes that express the enterovirus sensors melanoma differentiation-associated protein 5 (MDA5) and toll-like receptor- 3 (TLR3).

And, in a recent study using a reporter cell line infected with enterovirus strains isolated from T1D patients, immune transcriptome data supports the hypothesis of enterovirus-induced immune changes leading to the development of autoimmunity (9).

Infection by enteroviruses, such as coxsackieviruses, has been strongly associated with the autoimmune disease process T1D (2, 10). MDA5 and TLR3 are double-stranded RNA virus sensing proteins that specifically detect and protect from coxsackievirus infection and upon viral RNA detection, MDA5 and TLR3 activate and stimulate a cascade of anti-viral responses leading to the production of IFN-I (11, 12). Depending on their levels of expression and the timing and location of signaling, MDA5 and TLR3 can also protect from the onset of autoimmune diabetes following infection with diabetogenic viruses like coxsackieviruses and the pancreatropic RNA virus encephalomyocarditis virus strain D (EMCV-D) (1, 3, 12–15). In MDA5 heterozygous NOD mice, reduced expression of MDA5 induces a unique IFN-I signature with greater IFN-β and this protects mice from T1D after CB4 infection (13). Further, blocking IFN-α, but not IFN-β prevented T1D in the RIP-LCMV Tg model post LCMV infection (16).

Herein, we will show in greater detail, how changes in sensing of the same virus by MDA5 and TLR3 can lead to distinct signatures of interferon (INF)-α and IFN-ß resulting in different autoimmune diabetes disease outcomes. These findings add to the growing knowledge of interferonopathy as a contributor in enterovirus-driven autoimmunity (2, 3, 17) and highlight further, the importance of the local immune environment at the site of autoimmunity as an indicator of disease susceptibility.

## Materials and Methods

### Mice

NOD/ShiLtJ mice were purchased from The Jackson Laboratory (Bar Harbor, ME). MDA5^+/-^ mice were backcrossed from C57BL/6 MDA5^-/-^ mice onto the NOD background as previously described. We confirmed by SNP analysis (in house and by DartMouse, Lebanon, NH) that they carry the full complement of NOD *idd* alleles. More importantly, we confirmed that littermates to the backcrosses that were either heterozygous or wild-type for the MDA5^-/-^ alleles mice had the ability to develop spontaneous diabetes which is strongly indicative that the required susceptibility loci had crossed over. TLR3^-/-^ mice were obtained from The Jackson Laboratory (Bar Harbor, USA) and were backcrossed and maintained on the NOD/ShiLtJ mouse background. NOD TLR3^+/-^ and TLR3^+/+^ progeny were bred for use in experiments. Mice were maintained in the Modified Barrier Facility (Pharmaceutical Sciences Building, Vancouver, British Columbia) and kept in a pathogen-free environment. Diabetes incidence was monitored by non-fasting blood glucose measurements. Disease onset was determined by two consecutive blood glucose levels exceeding 300 mg/dL. Only pre-diabetic mice were used for experiments. All animal work was performed under strict accordance with the recommendations of the Canadian Council for Animal Care. The protocol was approved by the Animal Care Committee (ACC) of the University of British Columbia.

### Western Blotting

Mice were stimulated by intraperitoneal injection with 100μg of polyinosinic:polycytidylic acid (P1530, Sigma, St. Louis, MO). After 24 hours stimulation, spleens were isolated and homogenized by sonication and tissue homogenates were lysed with CellLytic MT Mammalian Tissue Lysis Reagent (Sigma, St. Louis, MO). Samples were separated on 10% sodium dodecyl sulfate-polyacrylamide gels, transferred to polyvinylidene fluoride membranes, blocked with Odyssey Blocking Buffer (LI-COR, Lincoln, NE) probed with monoclonal mouse anti-TLR3 (Novus Biologicals, Littleton CO) and polyclonal goat anti-tubulin Santa Cruz Biotech, Santa Cruz, CA) primary antibodies and IRDye 800CW and IRDye 680 RD secondary antibodies (LI-COR, Lincoln, NE). Membranes were scanned with the LI-COR Odyssey Scanner (LI-COR, Lincoln, NE). Protein was quantified using LI-COR Odyssey 3.0 Software.

### Virus

Ten-to 12-week old mice were infected intraperitoneally with sublethal doses of 400 plaque-forming units (PFUs) of CB4 Edwards strain 2 or coxsackievirus group B type 3 (CB3, Nancy Strain) diluted in DMEM. As there is no gender bias in CB4-mediated T1D, equal numbers of male and female mice were infected with CB4. Both male and female mice were infected with CB3. Virus stocks were prepared and free virus particles were detected from tissue homogenates by plaque assay as described previously (13).

### Immunohistochemical and Immunofluorescent Staining

Single cell-suspensions from pancreatic lymph nodes and spleens were restimulated for 4 hours at 37°C in Iscove’s modified Dulbecco’s medium containing 10% fetal bovine serum with 500ng/ml PMA, 10 ng/ml ionomycin and Golgi Plug (BD Biosciences). Cells were stained for surface and intracellular markers (**Table S2**), fixed, permeabilized, stained for inflammatory cytokines like γ-interferon (IFN-γ) and analyzed by flow cytometry. Cytokines IL-2, IL-4, IL-6, IL-10, IL-17, TNF-α, and IFN-γ were measured from serum days 0, 3 and 7 post-CB4 infection in a multiplexed format using a Cytometric Bead Array (mouse Th1/Th2/Th17 cytokine kit; BD Biosciences, Mississauga, ON). IFN-Is, IFN-α and-β were measured from serum by ELISA using VeriKine Mouse Interferon-α and β ELISA kits (PBL Interferon Source, Piscataway, NJ). All mAbs were purchased from eBiosciences (San Diego, CA) with the exception of Helios from BioLegend (San Diego, CA). Stained cells were analyzed by flow cytometry with the BD Biosciences LSR II (San Jose, CA) and Flow Jo vX.0.6 software (TreeStar, Ashland, OR).

### Reverse Transcription and Quantitative Real-Time PCR

Organs were removed and immediately snap frozen in TRIzol reagent (Life Technologies Inc, Burlington, ON). Tissues were weighed and organs were homogenized using QIAGEN stainless steel beads and TissueLyser II benchtop homogenizer at 19/s for 10 min. Total RNA was prepared with TRIzol reagent according to the manufacturer’s protocol (TRIzol, Life Technologies). RNA was quantified using a NanoDrop-ND-1000 (VERIFY) (Thermo Scientific, Wilmington, DE).

cDNA was prepared for 1μg of RNA using High Capacity cDNA Reverse Transcription Kit (Applied Biosystems, Foster City, CA) according to the manufacturer’s instructions. Reverse transcription-PCR was performed with the BioRad T-100 Thermal Cycler. cDNA was diluted with UltraPure™ DNase/RNase-Free Distilled Water (Life Technologies) and a final RNA concentration equivalent to 10μg/μl was used for real time (RT)-PCR. Gene expression for MDA5, IFN-α, IFN-β, TLR3 and GAPDH was quantified using the iQ™ SYBR^®^ Green Supermix (BioRad, Mississauga, ON) PCR amplification was performed in 384-well plates with the ABI 7900HT Fast Real-Time PCR System (Applied Biosystems). All samples from three independent experiments were evaluated in duplicate amplification reactions. mRNA expression was normalized to GAPDH. The comparative C_t_ method was used as previously described and data is shown as -Δ C_t_ and fold change of -Δ C_t_ relative to Wt (NOD) samples (18). Primers used in this study are listed in **Table S2**.

### Poly I:C Treatment

At days 3 and 5 post-infection with CB4, mice were stimulated by intraperitoneal injection with 100μg of polyinosinic:polycytidylic acid (P1530, Sigma, St. Louis, MO).

### Statistical Analysis

GraphPad Prism 6.0 software (GraphPad, San Diego, CA) using the Student t test (two-tailed distribution) and a P value <0.05 determined statistical significance. Serum cytokine concentrations were determined with FCAP Array Software (BD Biosciences, Mississauga, ON). Data are presented as means ± SEM.

## Results

To better understand how immune pathologies like IFN-I responses that result from MDA5 and TLR3 signaling influence T1D development, we challenged heterozygous NOD mice that retained MDA5 and TLR3 function with known diabetes and IFN-I inducers like coxsackievirus B4 (CB4).

**Figure 1:**
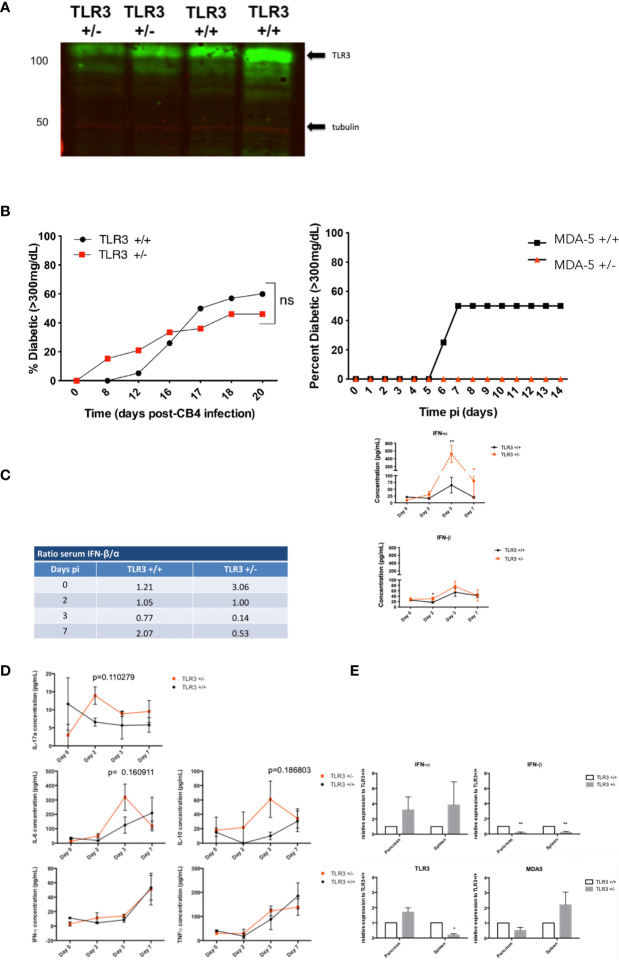
Diabetes incidence in TLR3^+/-^ and TLR3^+/+^ mice post- CB4 infection. **(A)** TLR3 expression in the spleens of TLR3 +/+, TLR3+/-, and TLR3-/- (not shown) mice that were stimulated with poly I:C as described in Materials and Methods. Twenty-four hours after stimulation, a reduction in TLR3 protein from TLR3 +/- spleens compared to TLR3 +/+ was confirmed by Western Blot. **(B)** TLR3 +/- (n = 13) and TLR3 +/+ (n = 20) (left) and MDA5+/- (n = 15) and MDA5 +/+ (n = 20)(right) were infected ip with 400 pfu CB4. Diabetes incidence was monitored up to 20 days post-infection. Two consecutive blood glucose levels greater than 300mg/dL determined diabetes incidence. Systemic and local inflammatory cytokines in TLR3^+/-^ and TLR3^+/+^ mice post-CB4 infection. **(C, D)** TLR3 +/- (n = 4-8) and TLR3 +/+ (n = 4-8) were infected by an intraperitoneal i.p. injection with 400 pfu CB4. IFN-α/β Inflammatory cytokine concentrations (pg/mL) were measured from sera by ELISA and IL-6, IL-10, IL-17a, IFN-γ, and TNF-α by FACS bead array at days 0, 2, 3 and 7 post-infection (pi). Ratios of the average IFN-β concentrations *versus* IFN-α (left) and the individual concentrations (right) **(C)** and averages of inflammatory cytokines **(D)** measured from sera at each time point are shown. **(E)** Relative mRNA expression levels of TLR3, MDA5, IFN-α and β from the spleen and pancreas of TLR3+/+ (n = 4-8) and TLR3+/- mice (n = 4-8) at day 3 post-CB4 infection were quantified by quantitative real time PCR and normalized to GAPDH. The comparative Ct method was used to calculate mean relative expression ± SEM against TLR3+/+ mice as described in *Materials and Methods* section. Data shown are from duplicate samples from two independent experiments. *p < 0.05, **p < 0.01 and ns, not significant.

Western blots confirmed a 50% reduction in MDA5 (13) and a 30% reduction in TLR3 expression (**Figure 1A**) in the spleens of our MDA5 and TLR3 heterozygous mice who were stimulated with an RNA mimetic polyinosinic:polycytidylic acid (poly I:C). Expression of MDA and TLR3 is below the level of detection in unstimulated mice (not shown). Complete deficiency in TLR3 expression (TLR3^-/-^) inhibits survival after CB4 infection (12), whereas a slight loss in TLR3 expression (TLR3^+/-^) retains sufficient signaling function for protection against CB4 (**Figure 1B**) and another coxsackievirus associated with T1D, coxsackievirus B1 (CB1, not shown). TLR3 expression, at reduced levels in TLR3^+/-^ mice, is not successful in protecting from CB4-induced T1D (**Figure 1B**) as up to 16 days post-infection, TLR3^+/-^ mice show a higher incidence of T1D compared to infected control wild type TLR3^+/+^ mice. Disease incidence in TLR3^+/-^ mice continued to rise to 50% by day 20 post-infection, just under the 60% disease incidence observed in TLR3^+/+^ mice. Though critical for survival, TLR3 expression does not contribute to protection from CB4-induced T1D, contrary to previous observations in MDA5^+/-^ mice, where a reduction in MDA5 increased survival and protected against the development of T1D after CB4 infection ^23^.

To examine antiviral responses in CB4 infected mice deficient in TLR3, inflammatory cytokines including IFN-I were measured in TLR3^+/-^ and TLR3^+/+^ mice post-infection at multiple time points using ELISA, cytometric bead array and real-time PCR (RT-PCR). Changes in TLR3 signaling induction of IFN-β compared to MDA5 signaling after infection with the same virus are further reflected in the ratio of sera IFN-β *versus* IFN-α levels. TLR3^+/-^ mice have three times the amount of IFN-β than IFN-α in the sera at baseline, prior to CB4 infection, compared to wt mice and by day 3 post infection, TLR3^+/-^ mice dramatically lose IFN-β production *versus* IFN-α in the sera (ratio of 0.14) compared to wt mice (ratio 0.71, **Figure 1C**).

This suggests that changes in TLR3 expression support the systemic production of IFN-α rather than IFN-β after CB4 infection, contrary to what we have previously observed for MDA5^+/-^ mice, where IFN-β responses are more significantly favored (13). Alterations in TLR3 signaling also induces the production of other inflammatory cytokines after CB4 infection (**Figure 1D** unlike negligible responses observed with changes in MDA5 signaling (13). Serum levels of IL-17a at day 2 post-infection and IL-6, and IL-10 at day 3 post-infection are increased in TLR3^+/-^ compared to CB4-infected wt mice (**Figure 1D**). In TLR3^+/-^ mice, IFN-β is significantly reduced and IFN-α is increased in both the pancreas and spleen (**Figure 1E**). The expression of IFN-I stimulators MDA5 and TLR3 is also tissue-specific. In CB4-infected NOD mice heterozygous for MDA5 (13) expression of both MDA5 and TLR3 is increased compared to wt NOD mice at day 3 post-infection, whereas in CB4-infected TLR3^+/-^ mice only TLR3 expression is increased compared to wt mice in the pancreas (**Figure 1E**). CB4 infection in TLR3^+/-^ mice also induces a significant reduction in TLR3 and increased MDA5 expression in the spleen (**Figure 1E**), opposite to infected MDA5^+/-^ mice that have decreased MDA5 and increased TLR3 expression in the spleen (13). Overall TLR3^+/-^ and MDA5^+/-^ mice have distinctive tissue specific expression of IFN-I inducers TLR3 and MDA5 and unique tissue-specific IFN-I responses after infection with the same virus.

**Figure 2:**
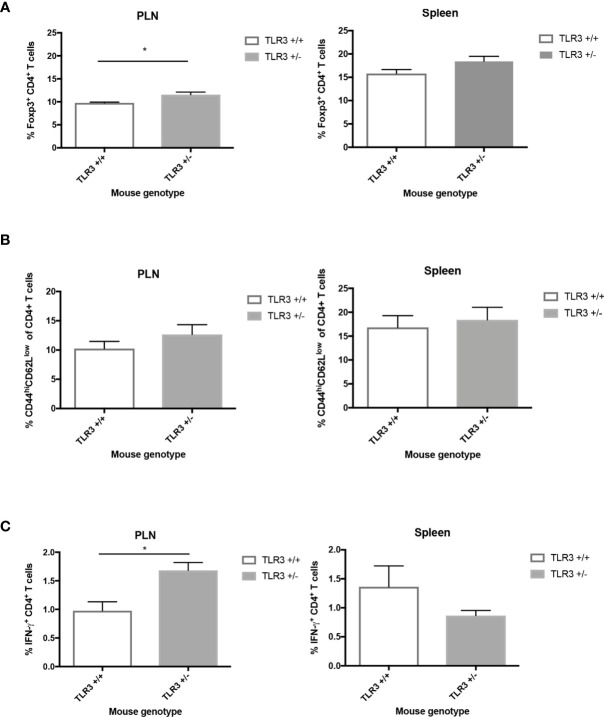
Regulatory and effector CD4+ T cells from the PLNs and spleen of TLR3^+/-^ and TLR3^+/+^ mice post-CB4 infection. **(A)** Regulatory T cells (Foxp3^+^ CD4^+^) and **(B, C)** effector T cells (CD44^hi^CD62^low^CD4^+^ and IFNγ-producing CD4^+^) were isolated from the pancreatic lymph nodes (PLNs) and spleens of TLR3+/+ (n = 5) and TLR3+/- mice (n = 5) at day 7 post CB4-infection and were stained with classical activation and maturation marker antibodies for FACS analysis. Results are shown as mean ± SEM of a representative from three independent experiments. *p < 0.05.

Since we know from previous studies (2, 10, 13, 19) that an imbalance in cellular immune responses, especially locally, can lead to the development of T1D following virus challenge, we investigated the regulatory and effector cell responses in the pancreatic lymph nodes and spleen of CB4-challenged mice. Similar to MDA5^+/-^ mice (13), at day 7 following CB4 infection in comparison to wt mice, TLR3^+/-^ mice have significantly increased regulatory Foxp3^+^ T cells in the pancreatic lymph nodes (PLNs) and in the spleen, while statistical significance was not achieved, there was a propensity for increased Tregs (**Figure 2A**). Though unlike CB4-infected MDA5^+/-^ mice that have a T cell response skewed towards protection from T1D, infected TLR3^+/-^ mice have significantly increased effector T cells in the PLN (IFN-γ- CD4^+^ T cells) and are not protected from T1D.

While statistical signficance was not achieved, in comparison to wt mice at 7 days PI, infected TLR3^+/-^ mice have a tendency for increased CD44^hi^CD62L^lo^ CD4^+^ effector T cells in the PLNs and spleen(**Figure 2B**). Statistically significant increased levels of IFN-γ-producing CD4^+^ T cells are observed in the PLNs compared to infected wt mice (**Figure 2C**). The increase in IFN-γ and adaptive responses at the site of autoimmunity is thus greatly apparent with changes in TLR3 expression and begs the question whether it is the receptor expression and signaling or the location of viral infection and subsequent interferon and T cell responses rather that influences susceptibility to disease. To address this potential spatial relevance in the function of interferon and T cell responses against viral infection, we turned to our MDA5 model and compared the inflammatory responses from two pancreatropic infections.

To test if MDA5 signaling within the pancreatic islets protects from T1D, we infected MDA5^+/-^ mice with the beta cell tropic- B4 strain and in another set of mice, the non-beta cell tropic virus B3 strain of coxsackievirus. In NOD mice, both the B4 and B3 strains of coxsackievirus infect and cause significant inflammatory pathology in the acinar tissue in the pancreas (19) though CB4 and not CB3 infects the pancreatic beta cells and induces T1D.

**Figure 3:**
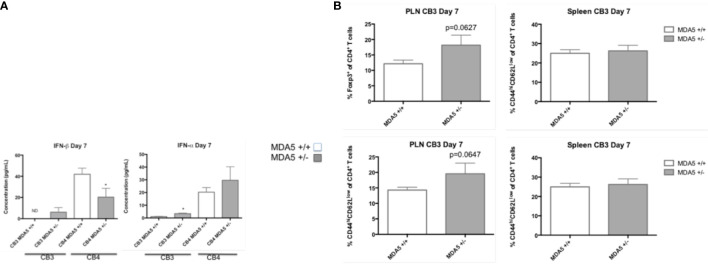
Serum levels of IFN-I and CD4^+^ T cell responses in MDA5^+/-^ and MDA5^+/+^ mice after CB3 and CB4 infection. **(A)** IFN-α/β Inflammatory cytokine concentrations (pg/mL) were measured from CB4 and CB3 infected MDA5^+/-^ (n = 7) and MDA5^+/+^ (n = 7) mice sera by ELISA. **(B)** Regulatory T cells (Foxp3^+^ CD4^+^) and effector T cells (CD44^hi^CD62^low^CD4^+^ and IFNγ-producing CD4^+^) were isolated from the pancreatic lymph nodes (PLNs) and spleens of CB3 infected MDA5^+/-^ (n = 5) and MDA5^+/+^ (n = 5) mice at day 7 post CB4-infection and were stained with classical activation and maturation marker antibodies for FACS analysis. Results are shown as mean ± SEM of a representative from three independent experiments. *p < 0.05.

Though CB3-infected MDA5^+/-^ mice have similar levels of inflammatory cytokines TNF-α, IFN-γ, IL-6, IL-17a, and IL-10 (data not shown) by day 7 following infection, CB3-infect MDA5^+/-^ mice have greater systemic levels of both IFN-α and IFN-β compared to CB3-infected wt mice (**Figure 3A**). CB4-infected MDA5^+/-^ mice also have increased systemic IFN-α at day 7 post-infection compared to infected wt mice, though they have a significant decrease in IFN-β (**Figure 3A**).

In MDA5^+/-^ mice, CB4 infection also results in increased regulatory Foxp3^+^ CD4^+^ T cells at the site of autoimmunity ^23^. Infection with the non-diabetogenic non-beta cell tropic virus CB3 in MDA5^+/-^ mice, however, induces a more inflammatory response with the increased presence of both regulatory Foxp3^+^ CD4^+^ T cells and effector CD44^hi^CD62L^lo^ CD4^+^ T cells at the site of autoimmunity, in the pancreatic lymph nodes (PLNs) (**Figure 3B**) compared to infected wt mice. And also unlike CB4-infected MDA5^+/-^ mice (13), CB3-infected MDA5^+/-^ mice have similar regulatory and effector CD4^+^ T cell responses in the spleen compared to infected wt mice (**Figure 3B**). With CB4 infection, effector T cells (CD4^+^ and CD8^+^) are significantly reduced in MDA5^+/-^ mice PLNs and spleen (13). These observations demonstrate that infection outside the islets with CB3, leads to systemic, and localized inflammatory responses. Infection within the islets, with CB4, induces regulatory, suppressive responses.

As humans encounter various pathogenic insults from their environment, it is likely that these pathogenic insults induce frequent bursts of IFN-I signaling as a result of innate immune responses. Consequently, innate responses and pathogen clearance may also frequently alter the IFN-I signature in the host and as such, alter immune homeostasis. To simulate alterations in immune homeostasis with frequent IFN-I production and to test whether the IFN-I signature and protective adaptive responses we observe in CB4-infected MDA5^+/-^ mice can be maintained despite additional bursts of IFN-I, we stimulated CB4-infected MDA5^+/-^ and MDA5^+/+^ mice 3 and 5 days post-CB4 infection with the dsRNA mimetic poly I:C.

**Figure 4:**
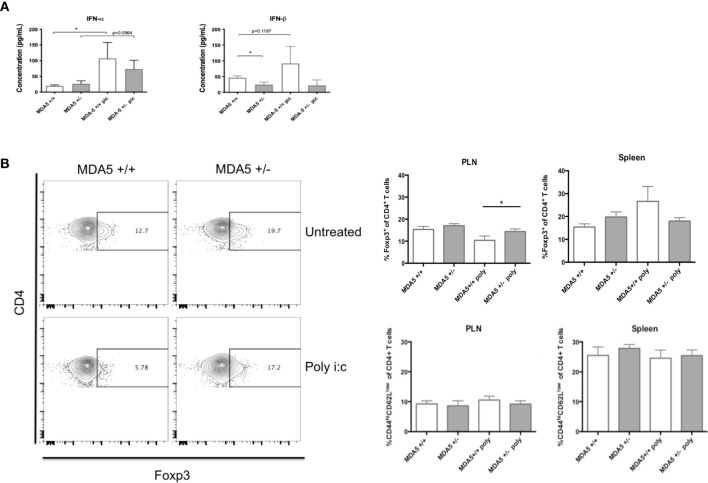
Systemic IFN-I levels, regulatory and effector CD4^+^ T cell responses in MDA5^+/-^ and MDA5^+/+^ mice after poly I:C stimulation and CB4 infection. **(A)** IFN-α/β Inflammatory cytokine concentrations (pg/mL) were measured from MDA5^+/-^ (n = 7) and MDA5^+/+^ (n = 7) mice sera at day 7 post-infection by ELISA. **(B)** Regulatory T cells (Foxp3^+^ CD4^+^) and effector T cells (CD44^hi^CD62^low^CD4^+^ and IFNγ-producing CD4^+^) were isolated from the pancreatic lymph nodes (PLNs) and spleens of poly I:C treated and untreated MDA5^+/-^ (n = 5) and MDA5^+/+^ (n = 5) mice at day 7 post CB4-infection and were stained with classical activation and maturation marker antibodies for FACS analysis. Results are shown as mean ± SEM of a representative from three independent experiments. GraphPad Prism 6.0 software (GraphPad, San Diego, CA) using the Student t test (two-tailed distribution) and a P value < 0.05 determined statistical significance *p < 0.05.

Contrary to what was expected, a reduction in MDA5 impressively held a unique IFN-I signature despite additional IFN-I stimuli following CB4 infection. At day 7 post-infection, MDA5^+/-^ mice treated with poly I:C at days 3 and 5, maintain lower IFN-β levels similarly to unstimulated MDA5^+/-^ mice. Poly I:C stimulated MDA5^+/-^ mice do, however, show an increase in IFN-α production by day 7 post-infection compared to unstimulated CB4-infected MDA5^+/-^ mice (**Figure 4A**). Poly I:C treatment holds a greater effect on MDA5^+/+^ mice, where both IFN-α and β serum levels are increased at day 7 post-infection compared to untreated CB4-infected wt mice (**Figure 4A**).

To further study the immune consequences of additional IFN-I stimulation in CB4-infected MDA5^+/-^ mice, we examined the expression of significant APC activation markers CD40, CD80 (data not shown) and CD86 in poly I:C treated, CB4-infected MDA5^+/-^ mice. After poly I:C treatment and seven days of CB4 infection, APCs (CD11b^+^CD11c^+^, CD11b^+^CD11c^-^) isolated from PLNs and spleens of MDA5^+/-^ mice express similar levels of CD40 and CD86 compared to untreated CB4-infected MDA5^+/-^ mice (**Figure S2**). With insignificant APC activation, it is also no surprise that pro-inflammatory cytokine production is also insignificant at day 7 infection (data not shown). Further, T cell responses in poly I:C treated and CB4-infected MDA5^+/-^ remain similar with the response in untreated CB4-infected MDA5^+/-^ mice while the number of Foxp3^+^ CD4^+^ regulatory T cells remained elevated in the PLNs relative to the number of effector CD44^hi^ CD62L^lo^ CD4^+^ T cells (**Figure 4B**). This suggests that APC activation and subsequent T cell polarization in MDA5^+/-^ is unaffected by additional IFN-I stimulus.

Injections of poly I:C have a more dramatic effect on CB4-infected MDA5^+/+^ mice. Although CD86 expression remained unchanged in CB4-infected MDA5^+/+^ mice following poly I:C treatment, CD40 expression on CD11b^+^CD11c^+^ cells was decreased (**Figure S2**), regulatory T cells were slightly increased in the spleens (**Figure 4B**) and notably, regulatory T cells were significantly decreased in the PLNs (**Figure 4B**) compared to untreated, infected MDA5^+/+^ mice. These results emphasize the strength of the regulatory protective phenotype observed in MDA5^+/-^ mice to remain at the site of autoimmunity, which is not observed in MDA5^+/+^ mice. Despite the additional poly I:C treatment and potential for greater IFN-I, by day 7 post-infection reduced MDA5 signaling still results in a lower IFN-I level with greater regulatory T cells in the PLNs, at the site of autoimmunity.

## Discussion

Establishing a balance between immune activation and immune protection is essential in an already fragile autoimmune-susceptible state. Double stranded RNA sensors like MDA5 and TLR3 are critical in recognizing specific RNA viruses and inducing an inflammatory IFN-I antiviral response to help clear the invading RNA virus. Here, we have provided evidence of a unique and protective IFN-I and regulatory T cell signature induced with a reduction in MDA5 that is specific to changes in MDA5 signaling and not with a reduction in TLR3 signaling. Rather, the two viral sensors signal co-operatively, similar to a thermostat or counter-balance to achieve a range of immune responses.

Although TLR3 signaling is critical for survival against CB4 in NOD mice (12), we observed that IFN-I and adaptive responses produced as a result from TLR3 signaling to clear the virus are not similarly compatible with protection from autoimmunity. CB4-infected TLR3^+/-^ mice have skewed IFN-I responses that favor increases in IFN-α and increases in effector T cells at the site of autoimmunity that ultimately do not protect from T1D.

In diabetes resistant C57BL/6 mice, MDA5 and TLR3 signaling are both required to prevent diabetes following infection with a pancreatropic virus encephalomyocarditis virus strain D (EMCV-D). EMCV-D, however, induces diabetes through the direct destruction of β cells rather than T cell–mediated autoimmunity that we observe with CB4 infection. Interestingly, MDA5 and TLR3 exert different IFN-I response kinetics following EMCV-D infection in C57BL/6 mice, with IFN-I responses detected in MDA5^-/-^ at 15 hours post-infection and at a later time in TLR3^-/-^ mice (15). The potential cooperative role in IFN-I signaling seems to be specific to the EMCV-D infection model in diabetes resistant C57BL/6 mice. With our CB4-infection model in diabetes susceptible NOD mice we observe distinct IFN-I responses from MDA5 and TLR3 signaling and we do not see a cooperative mechanism from either receptor attempting to compensate for the lack in expression of the other in our heterozygous or knockout (not shown) mice. From what we have demonstrated with our model, TLR3 signaling is more important for anti-viral responses and rather the IFN-I signature and adaptive responses from MDA5 signaling, though still critical for the anti-viral response, are significant in protecting from autoreactive responses.

The location of viral infection and thus the location of MDA5 signaling in response to viral infection is also important in considering anti-viral immune responses such as IFN-I that could exacerbate and activate pre-existing autoreactive responses. IFN-I are primarily produced by the β cells in the pancreas with coxsackievirus infection and intraislet IFN-I production has shown to prevent CB4 replication (20, 21). In T1D patients, a unique IFN-I signature precedes islet autoimmunity and expression of interferon-stimulated genes is exacerbated in insulitic islets (6). The nature of the IFN-I response within the islets is therefore a critical component in the pathology that leads to autoimmunity in T1D.

We previously demonstrated that CB4-infected MDA5^+/-^ mice have tissue-specific and unique systemic IFN-I and adaptive responses that ultimately lead to protection from T1D (13). Here we sought to determine whether MDA5 signaling and anti-viral IFN-I responses within the islets would contribute to the protective phenotype. After challenging MDA5^+/-^ mice with the B3 strain of coxsackievirus that infects and causes significant inflammatory acinar tissue pathology outside of the islets, in comparison to mice challenged with the β- cell tropic virus CB4 we determined whether viral islet tropism could change anti-viral and autoreactive responses. We observed that it is likely that the location of virus infection in MDA5^+/-^ mice that alters IFN-I and adaptive responses. With CB3 infection, we observed in MDA5^+/-^ mice, an increase in systemic IFN-I levels and localized numbers of effector T cells at the site of autoimmunity, similarly to CB3-infected MDA5^+/+^ mice and in contrast to the phenotype observed with CB4 infection.

MDA5 signaling of CB4 and likely other β-cell tropic viruses followed by the presentation of β-cell antigens is therefore necessary to maintain a unique IFN-I signature and regulatory T cell responses at the site of autoimmunity that can ultimately protect from T1D. By regulating the induction of IFN-I at the site of infection, virus infection creates a local site of interferonopathy leading to altered T cell responses, loss of T cell regulation and induction of autoimmune diabetes. As CB4-MDA5^+/-^ mice have a burst in IFN-β shortly after infection that subsides by day 7 post-infection, it is likely that controlled, local, IFN-β signaling at the site of the islet β cell, leads to protection from exacerbated autoreactive responses and ensuing autoimmunity. Studies investigating the role of localized regulation of IFN-I within the islets could establish a new therapeutic avenue for preventing the autoimmune process and susceptibility to T1D.

In addition to considering the localized islet environment, it is also important to consider the environment of the host. As individuals are exposed to multiple environmental factors including viruses, they are exposed to frequent sources of IFN-I stimuli. Frequent bursts of IFN-I, depending on the location and susceptibility of the host, could lead to abrogated immune responses and lead to autoimmunity. To reproduce an environment where the host is exposed to frequent IFN-I stimulation and determine whether additional IFN-I responses are capable in offsetting an existing protective IFN-I signature, we infected MDA5^+/-^ and MDA5^+/+^ controls with CB4 and at days 3 and 5 post-infection, injected CB4-infected mice with the artificial dsRNA mimetic poly I:C.

In NOD mice, at a dose of 5µg/g body weight, poly I:C protects from T1D (22) and in NOD BDC2.5 transgenic mice that do not develop spontaneous diabetes, a similar poly I:C dose is not capable of inducing resting autoreactive memory T cells and diabetes (19). Though poly I:C signals MDA5 and activates IFN-I responses, the dsRNA mimetic does not induce β cell damage directly like β -cell tropic agents such as CB4. In asking whether recurrent IFN-I responses induced by subsequent treatments of poly I:C following CB4 infection could offset an existing IFN-I and immunoregulatory phenotype in MDA5^+/-^ mice, we did not expect and were not surprised to observe that poly I:C treatment did not further accelerate disease onset in our mice following CB4 infection. Instead, it might be expected that in addition to an already existing breakdown in tolerance, a second insult inducing IFN-I, such as poly I:C treatment, could regress tolerogenic mechanisms and progress disease pathogenesis to autoimmunity. As such, we expected that supplemental IFN-I stimulation with poly I:C post-CB4 infection would abrogate the IFN-I signature with higher systemic IFN-I levels and polarized T cell responses typically observed in CB4-infected, untreated MDA5^+/-^ mice would shift to effector rather than regulatory CD4+ T cells dominating in the PLNs. To our delight, we observed that despite the additional IFN-I stimulation with CB4 infection and two doses of poly I:C, MDA5^+/-^ mice maintain IFN-I and immunoregulatory responses similar to the protective phenotype observed in untreated CB4-infected MDA5^+/-^ mice. Though poly I:C is known to stimulate APC maturation and polarize T cell responses in certain susceptible models, we did not observe changes in the expression of classic APC markers CD40, CD80 or CD86 or changes in T cell responses in poly I:C treated CB4-infected MDA5^+/-^ mice from the untreated phenotype.

The reduction and changes in MDA5 signaling at the site of autoimmunity in MDA5^+/-^ mice likely allows for a balanced anti-viral and immunoregulatory adaptive response in the events of exposure to frequent IFN-I stimulation. With low-dose injections (0.05µg/g body weight) in the BioBreeding (BB), diabetes-resistant rat model, poly I:C protects rather than accelerates disease, mostly attributing to the induction of suppressor T cell activity (23). With the B7.1 C57BL/6 model, where the B7.1 costimulatory molecule is expressed in islets, the level of poly I:C–induced IFN-α determines the frequency and timing of diabetes onset, where higher levels of IFN-α coincide with accelerated, earlier onset of T1D (24). These models along with our MDA5^+/-^ model suggest that low level IFN-I signaling, as a result of a reduction in MDA5, can help avoid and regulate rather than activate autoreactive responses in a genetically susceptible host. Local anti-viral IFN-I responses in the pancreas likely create a site of interferonopathy that shifts the balance from immunoprotective to fully regressed immunosusceptible and prone to autoimmunity.

## Conclusions

With our MDA5^+/-^ and TLR3^+/-^ CB4-infection models, we have identified, using a virus clinically linked to T1D, how changes in MDA5 and not TLR3 signaling are critical in producing an IFN-I response and subsequent adaptive responses that protect from T1D. By challenging MDA5^+/-^ mice with different strains of coxsackieviruses we have teased out the location specific importance of IFN-I signaling and the interferonopathy within the pancreas that changes with a reduction in MDA5 and allows for IFN-I and T cell responses in favor of protection from T1D. Conversely, challenging TLR3^+/-^ mice resulted in the opposite effect by retaining an IFN-I response that resolves to T1D. This further dissects the IFNα/β responses to separate sensors demonstrating a need, and not a redundancy, for both TLR3 and MDA5 to sense and respond differentially to infection. Further, in treating MDA5^+/-^ mice with poly I:C, an additional IFN-I inducer, following CB4 infection, we simulated the environmental context in which humans are exposed to multiple IFN-I stimuli, and demonstrated the strength of the protective phenotype that results with a reduction in MDA5 signaling in withstanding additional IFN-I chaos. MDA5 serves as a cellular protector from viral invasion and as such, with inter-individual variability, MDA5 activity will change with variations at the genetic and immunological expression level. Establishing low level IFN-I signaling from MDA5 may be host specific as we see with IFIH1 heterozygous individuals that carry the protective allele. Regulating IFN-I levels to evade interferonopathies whether systemic or organ-specific still remains an effective strategy in evading autoimmunity as we have demonstrated using an autoimmune diabetes model. We have not demonstrated another way to prevent T1D in the NOD mouse, rather we believe this work has provided compounding evidence for a specific control of IFN-I to drive a myriad of responses ranging from virus clearance to onset of autoimmune diabetes.

## Data Availability Statement

The original contributions presented in the study are included in the article/**Supplementary Material**. Further inquiries can be directed to the corresponding author.

## Ethics Statement

The animal study was reviewed and approved by UBC Animal Care Committee.

## Author Contributions

Conceptualization, PL and MH. Methodology, PL, IS, and MH. Software, X.X. Validation, PL, IS, and MH. Formal analysis, PL and MH. Investigation, PL and MH. Resources, MH. Data curation, PL. Writing—original draft preparation, PL. Writing—review and editing, PL and MH. Supervision, MH. Funding acquisition, MH. All authors contributed to the article and approved the submitted version.

## Funding

This research was funded by Juvenile Diabetes Research Foundation and Canadian Institutes of Health Research.

## Conflict of Interest

The authors declare that the research was conducted in the absence of any commercial or financial relationships that could be construed as a potential conflict of interest.

## Publisher’s Note

All claims expressed in this article are solely those of the authors and do not necessarily represent those of their affiliated organizations, or those of the publisher, the editors and the reviewers. Any product that may be evaluated in this article, or claim that may be made by its manufacturer, is not guaranteed or endorsed by the publisher.
