# Supplementary material for: Changes in MDA5 and TLR3 Sensing of the Same Diabetogenic Virus Result in Different Autoimmune Disease Outcomes
Source: Front Immunol. 2021 Nov 5;12:751341. doi: 10.3389/fimmu.2021.751341 (PMC8602094; doi:10.3389/fimmu.2021.751341)
Supplement: Supplementary file 1 [file Image_1.pdf]

Figure S1

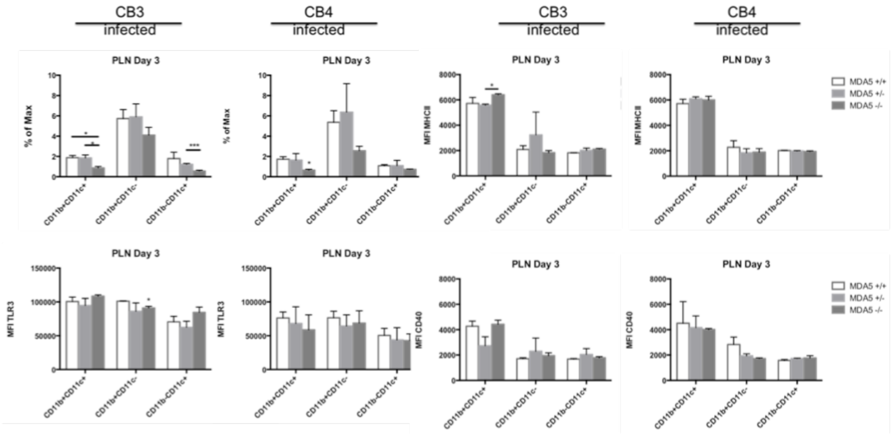

Figure S1. Antigen presenting cell activation and responses in MDA5$^{+/-}$ and MDA5$^{+/+}$ mice post-CB3 and CB4 infection.
