# Supplementary material for: Changes in MDA5 and TLR3 Sensing of the Same Diabetogenic Virus Result in Different Autoimmune Disease Outcomes
Source: Front Immunol. 2021 Nov 5;12:751341. doi: 10.3389/fimmu.2021.751341 (PMC8602094; doi:10.3389/fimmu.2021.751341)
Supplement: Supplementary file 2 [file Image_2.pdf]

Figure S2

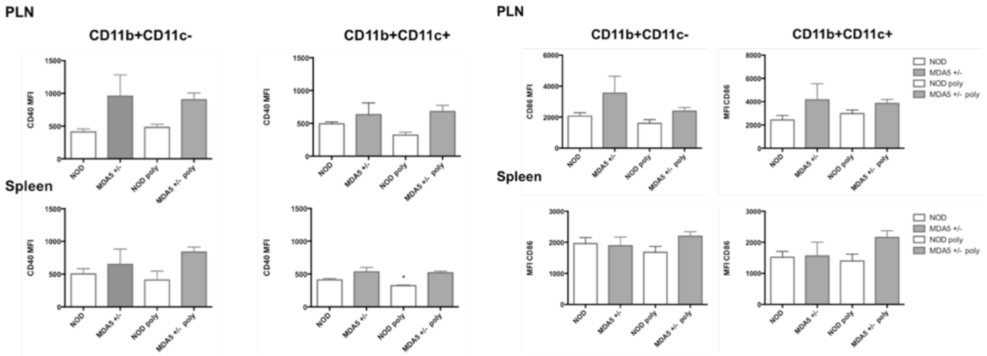

Figure S2. Antigen presenting cell activation in MDA5$^{+/-}$ and MDA5$^{+/+}$ mice after poly I:C stimulation and CB4 infection.
