# Supplementary material for: Changes in MDA5 and TLR3 Sensing of the Same Diabetogenic Virus Result in Different Autoimmune Disease Outcomes
Source: Front Immunol. 2021 Nov 5;12:751341. doi: 10.3389/fimmu.2021.751341 (PMC8602094; doi:10.3389/fimmu.2021.751341)
Supplement: Supplementary file 3 [file Table_1.pdf]

Table S1. Immunofluorescent staining.

Location	Marker	mAB
**Surface**	CD4	Clone L3T4
	CD8	53-6.7
	C25	Clone PC61
	CD11b	Clone M1/70
	CD11c	Clone HL3
	CD44	Clone IM7
	CD40	Clone 3/23
	CD62L	Clone MEL-14
	CD80	Clone 16-10A1
	CD86	Clone GL1
**Intracellular**	Foxp3	Clone FJK-16s
	Helios	Clone 22F6
	TLR3	Clone 40C1285.6
	MDA5	Abcam ab69983
	IFN-γ	XMG1.2
	IL-17	Clone TC11- 18H10.1
