# Supplementary material for: Changes in MDA5 and TLR3 Sensing of the Same Diabetogenic Virus Result in Different Autoimmune Disease Outcomes
Source: Front Immunol. 2021 Nov 5;12:751341. doi: 10.3389/fimmu.2021.751341 (PMC8602094; doi:10.3389/fimmu.2021.751341)
Supplement: Supplementary file 4 [file Table_2.pdf]

Table S2. Reverse transcription and quantitative real-time PCR primers

**Mouse Gene Target**	**Sequence**
**MDA5 forward**	5′- GTGATGACGAGGCCAGCAGTTG-3′
**MDA5 reverse**	5′- ATTCATCCGTTTCGTCCAGTTTCA-3′
**IFN-β forward**	5′- GCACTGGGTGGAATGAGACTATTG-3′
**IFN-β reverse**	5′- TTCTGAGGCATCAACTGACAGGTC-3′
**IFN-α forward**	5′- TGATGAGCTACTGGTCAGC-3′
**IFN-α reverse**	5′- GATCTCTTAGCACAAGGATGGC-3′
**TLR3 forward**	5′- GAGAGAGATTCTGGATGCTTGTGTTTG-3′
**TLR3 reverse**	5′- TGTAGACCATGTAGTTGAGGTCA-3′
**GAPDH forward**	5′- AGGTCGGTGTGAACGGATTTG-3′
**GAPDH reverse**	5′- TGTAGACCATGTAGTTGAGGTCA-3′
